# The Effect of Perceived Risks on the Demand for Vaccination: Results from a Discrete Choice Experiment

**DOI:** 10.1371/journal.pone.0054149

**Published:** 2013-02-08

**Authors:** Md Z. Sadique, Nancy Devlin, William J. Edmunds, David Parkin

**Affiliations:** 1 Department of Health Services Research and Policy, London School of Hygiene & Tropical Medicine, London, United Kingdom; 2 Office of Health Economics, London, United Kingdom; 3 City Health Economics Centre, City University, London, United Kingdom; 4 Department of Infectious Disease Epidemiology, London School of Hygiene & Tropical Medicine, London, United Kingdom; 5 NHS South East Coast, Surrey, United Kingdom; Melbourne School of Population Health, Australia

## Abstract

The demand for vaccination against infectious diseases involves a choice between vaccinating and not vaccinating, in which there is a trade-off between the benefits and costs of each option. The aim of this paper is to investigate these trade-offs and to estimate how the perceived prevalence and severity of both the disease against which the vaccine is given and any vaccine associated adverse events (VAAE) might affect demand. A Discrete Choice Experiment (DCE) was used to elicit stated preferences from a representative sample of 369 UK mothers of children below 5 years of age, for three hypothetical vaccines. Cost was included as an attribute, which enabled estimation of the willingness to pay for different vaccines having differing levels of the probability of occurrence and severity of both the infection and VAAE. The results suggest that the severity of the health effects associated with both the diseases and VAAEs exert an important influence on the demand for vaccination, whereas the probability of these events occurring was not a significant predictor. This has important implications for public health policy, which has tended to focus on the *probability* of these health effects as the main influence on decision making. Our results also suggest that anticipated regrets about the consequences of making the wrong decision also exert an influence on demand.

## Introduction

Vaccination programmes have succeeded in reducing the adverse health effects of many diseases. However, in recent years people have become increasingly aware of the possible adverse side effects of vaccines. Declining vaccine uptake rates have been observed in many developed countries, leading to concern that epidemics may re-emerge. Policy makers are therefore interested in understanding the factors associated with acceptance of vaccination. Understanding the determinants of demand can help to improve predictions of vaccine uptake rates, identify effective policy interventions to increase demand and facilitate the economic evaluation of policy measures.

In choosing whether or not to vaccinate, people are affected by perceived *risks*, by which we mean exposure to a factor that may lead to impairment in their health. These risks comprise two elements: the probability of an undesired outcome, impaired health, and the magnitude of that outcome, the extent to which health is impaired. Both of these elements are relevant to both of the alternatives in a choice about vaccination – to vaccinate or not to vaccinate. For example, there is a probability of disease if the choice is to remain unvaccinated. A greater perceived probability of infection is likely to have a positive effect on the demand for vaccination against it. But there is also a probability of vaccine associated adverse events (VAAE), which will have a negative effect on vaccine demand. The nature of the overall risks may be quite different for different common infectious diseases. The probability of VAAE may be small, but the effects may be, or perceived to be, severe and permanent. The probability of infection if unvaccinated may be much higher, but the impact of the resulting disease may be, or perceived to be, temporary and minor.

Empirical analyses of the demand for vaccination have generally relied on observed real-world choices in the light of actual risks that were known by experts when those choices were made [1]. However it is possible that the risks actually being considered by people making these choices may differ from the real risks, reflecting the fact that they may gather information from various informal and formal sources of varying accuracy and relevance. In such circumstances, stated preference techniques for eliciting preferences have an advantage, since they allow the investigation of variables that are not observable from real-world data but may be influential in decision making.

This paper reports on a Discrete Choice Experiment (DCE) undertaken to investigate these issues, based on choices by mothers about three vaccines that might be given to their children. The DCE method is based on the premise that how people value a good or service can be evaluated by examining the nature and level of the attributes that the good or service has [2]–[5].

### Theoretical Considerations

Among the theories examining factors that contribute to decisions to vaccinate the Health Belief model has been the most widely applied [6]. The Health Belief model proposes that the decision to vaccinate is a function of perceived susceptibility to and severity of disease as well as concern about vaccine benefits and risks [7]–[10]. The perceived susceptibility to disease can be described as the subjective perceived risk (or probability) of contracting a disease [8]. The perceived severity of disease is the subjective feeling concerning the seriousness of disease including health and social consequences. The perception of benefit versus costs is the evaluation of the effectiveness of alternative actions that can be taken to reduce the disease threat [8]. The choice of vaccination is a decision under uncertainty, and choice is guided by balancing costs and benefits associated with vaccination versus non-vaccination. Sadique *et al* [11] hypothesised that the perceived relative risk of infection compared to that of VAAE has a threshold below which a person will prefer to remain exposed to the infectious disease and above which they will choose vaccination. This provides an intuitively plausible explanation for declining vaccine rates in developed countries. Vaccines have largely eliminated the threat of many serious childhood diseases, while concerns regarding alleged VAAE have increased. For many people, the perceived relative risk has fallen below the threshold, lowering the propensity to vaccinate.

This way of viewing vaccination decisions suggests the following hypotheses. First, the higher the perceived risk of VAAE, the lower the demand for vaccination is. Perceived risk is influenced by the probability and severity of VAAE. Secondly, the higher the perceived risk of infection is, influenced by the probability of infection and the severity of health effects resulting from infection, the higher the demand for vaccination is.

An obvious way to view the vaccination choice is that people maximise their expected utility, so that they demand vaccination when the expected gain from the lower risk of infection exceeds the expected gain from not being exposed to VAAE. But what if the choice is *ex post* wrong, in the sense that vaccination actually leads to VAAE or non-vaccination leads to infection? The possibility of *ex post* non-realisation of *ex ante* expectation can bring a sense of loss, or regret, that may also be anticipated *ex ante* [12]. Regret is a negative, cognitively based emotion that we experience when realising or imagining that our present situation would have been better, had we acted differently [13]. Regret theory suggests that decision-makers when making decisions under uncertainty optimise the expected value of a “modified” utility where expected utilities are modified by feelings of regret if things turn out worse than they would have done under the other option, or by rejoicing if things turn out better [12]. Using this insight, Sadique *et al* [11] suggested the following hypotheses. First, the higher the anticipated regret from VAAE, the lower the propensity to demand vaccination. Secondly, the higher the anticipated regret from infection if not vaccinated, the higher the propensity to demand vaccination.

In the case of childhood vaccination, parents act as agents for their child by weighing up the benefits and the costs from their decision to vaccinate or not, choosing the option that maximises their expected utility, or minimises their regret. In doing so, parents confront trade-offs between the risks that their child faces. These trade-offs are not confined to the consideration of risk; the decision to vaccinate is also contingent on other factors, such as the cost of obtaining vaccination.

## Data and Methods

A DCE was used to investigate the influence of risk perceptions, health impacts, and costs on the stated vaccination choices of mothers of young children. The study involved respondents being given a choice between two alternatives – ‘to vaccinate’ and ‘not to vaccinate’ – without the option to remain undecided between the two. The DCE method requires the identification of attributes of the objects of choice, in this case vaccination and non-vaccination, and the construction of choice scenarios involving different combinations of different levels of these attributes.

We chose to create choice scenarios about artificial rather than real vaccines. The reason for this was to avoid any pre-existing bias and to ensure that we had a wide range of levels for the attributes. The vaccines are based on real diseases: rotavirus, a common, but usually mild infection; invasive pneumococcal disease (IPD), a severe but rare disease; and non-invasive pneumococcal disease (NIPD), a disease with moderate incidence and severity. These were selected because of the contrasting nature of the risks associated with them and because vaccines for these were being considered for introduction into national immunisation schedules at the time of the study. The attributes and levels used were loosely based on the severity and probability of these diseases and the adverse events that may be associated with the vaccines for them.

The selection of attributes and their levels was informed by a review of the literature, which identified key influences on vaccination decisions. Risk perception is linked to vaccination behaviours [1], [14]–[17]. Relevant considerations include both the severity of health effects and the probability that they will be experienced [14], which led us to included both in our measure of risk perception. We identified three types of attributes likely to be important to vaccination choices: the price of the vaccine; the probability of the occurrence of health problems, and the severity of health problems. Including the price of the vaccination allows us to generate estimates of willingness to pay (WTP) for desirable attributes and willingness to accept (WTA) for undesirable attributes [18], [19]. The inclusion of a cost (or price) attribute in DCE makes it possible to indirectly obtain the respondent’s WTP for the attribute. This is one of the advantages of DCE where WTP estimates are obtained indirectly from the revealed preferences rather than asking WTP questions directly. However, the inclusion of a cost attribute, particularly within collectively funded healthcare systems can be challenging where individuals are not used to paying for a service or a good at the point of consumption. Some evidence suggests that the levels of the cost attribute can affect the estimates [20], but there is no evidence to suggest that including a cost attribute in a DCE leads to change in preference compared to that without a cost attribute [21].

Since the decision to vaccinate is modelled as a choice between vaccinating and not vaccinating, each attribute is divided into two groups. There are two types of probability of occurrence – the probability of infection and the probability of VAAE - and two types of severity of health consequences – the severity of infection, and severity of VAAE. Similarly, there are costs associated with a decision to vaccinate, and the cost of infection associated with a decision not to vaccinate. However, we only included the out-of-pocket price of vaccination, which is of course zero for the non-vaccination option. We assume that other relevant perceived costs, those resulting from the consequences of the choice, are taken into account in assessing the importance of the levels of severity of VAAE and infection.

The levels for the probability and severity attributes were chosen so that their description differed clearly and also broadly reflected the actual probabilities and severities of the diseases. The probabilities were generally inflated for the risks of vaccine associated side effects – otherwise the risks being described would have been extremely small. For each vaccine, three levels of severity and probability are presented. These are referred to as low, medium and high, although the descriptors for these levels differ between vaccines, reflecting the different characteristics of each disease. The attributes and levels used are reported in supporting Table S1.

**Figure 1:**
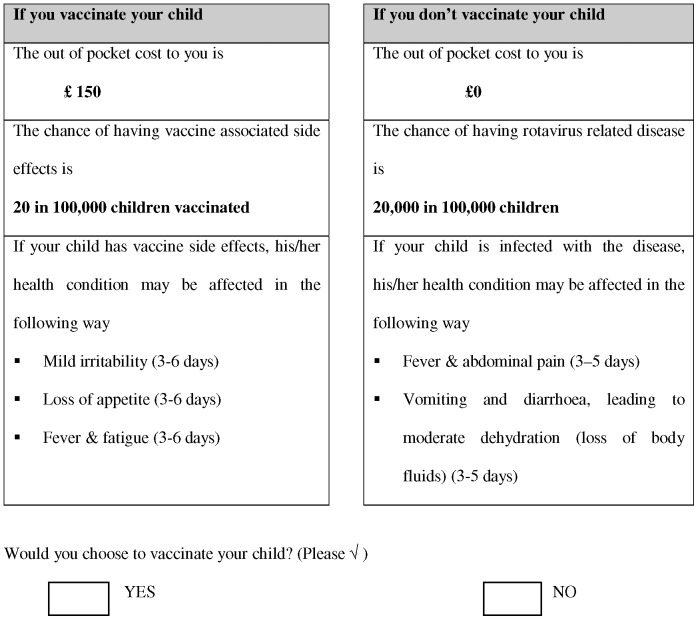
Sample choice set.

The combination of three attributes with three levels each resulted in 27(3^3^) choice sets for each of the two options, vaccination and non-vaccination. However, a fractional factorial design method [22] requires 9 choice sets for each option, which were combined into a single choice – vaccinate or not - using a fold over design. Because there are three diseases, each respondent would potentially face 27 different choice sets. A typical choice set is shown in Figure 1. Respondents were presented with choice sets and for each were asked their preferred decision.

A pilot study was carried out on a small convenience sample of mothers (n = 15) to test whether or not respondents could plausibly complete the DCE questionnaire and provide meaningful responses, to determine the best ways of expressing the attributes and to determine the numbers of attributes and levels. The pilot study enabled us to design the choice experiment so that it minimised task complexity and helped us to decide how best to present the probabilities and to frame questions on regret. However, it was clear from the pilot study that it was not feasible to ask participants to consider this number of choice sets. We therefore constructed 3 different versions of the questionnaire, each of which contained 9 choice sets, 3 for each disease.

**Figure 2:**
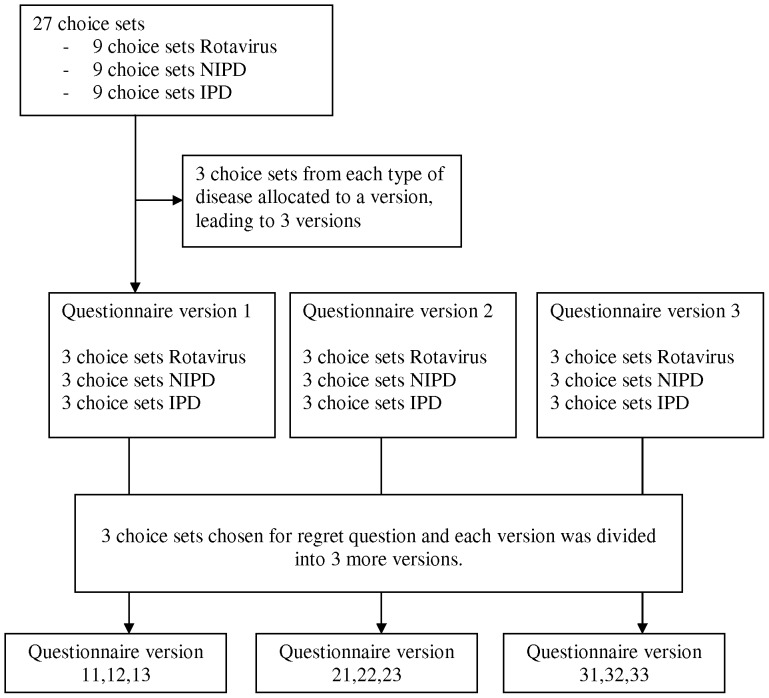
Flow diagram of questionnaire.

Each version of the questionnaire included a further 3 choice sets, randomly taking one choice set from each disease from the 9 choice sets that were initially presented, to which a question about regret was included. Each of the 3 initial versions of the questionnaire therefore has 3 variants, so that there were 9 different versions of the questionnaire. A flow of diagram of questionnaire allocation is given in Figure 2.

The regret questions assessed the strength of the emotions mothers anticipated that they might experience if their vaccination decision had adverse outcomes. On the basis of the attributes and levels in the corresponding choice set for which respondents had already indicated their preference, respondents were asked to indicate how they would experience a particular state of emotion on a 0–10 scale if their vaccination decision were to turn out badly, where 0 means *I would not experience this at all* and 10 means *I would experience this a lot*. This scale was based on that used in previous studies [16], [23], [24]. Respondents were asked these two questions: “If you have decided to **vaccinate**, how likely are you to regret this decision given that there is some chance that your child may experience vaccine associated side effects as described above?”; and “If you have decided to **not vaccinate**, how likely are you to regret this decision given that there is some chance that your child may experience disease as described above?”.

One more choice set was added to every questionnaire in which the attributes for vaccination are set at the best level and the attributes for non-vaccination are set at the worst level. This choice set was included as an extreme case where utility from the vaccination decision should be maximised and utility from non-vaccination should be minimised. The intention was to obtain information on how participants would respond to an extreme scenario, which might indicate that they have either extreme or irrational preferences.

To summarise, each respondent was asked to make 13 choices: 3 choice sets for each of the 3 diseases plus 3 choice sets including regret and one designed to test their choice under circumstances where vaccination is most likely to be attractive.

Respondents were also asked to evaluate the health states that appeared in the choice sets. They were first asked to rank the health states from best to worst. They then gave a rating of the health states from 0, meaning the worst imaginable health state, to 100, meaning the best imaginable health state, using a Visual Analogue Scale (VAS) similar to the EuroQol Group’s EQ-VAS [25]. The choice sets covered 15 different health states, but in order to minimise respondent burden each respondent were asked to evaluate only a subset of these health states in their corresponding questionnaire. Six out of a possible 15 health states were allocated to different versions of the questionnaire. A copy of the questionnaire is available on request.

A survey of mothers who had at least one child under the age of 5 years was conducted in January 2007. These were considered to be the group to whom the survey is most relevant. Approval for the study was obtained from the City University Research Ethics Committee. Verbal consent of respondents was obtained. A detailed explanatory statement was given to respondents describing the study, which highlighted that their participation was voluntary and no identifiable personal data would be collected. The sample was recruited and data were collected by a market research agency, using a nationally representative sampling frame across England and boosted in Wales to ensure that we have proportional regional quotas. Interviews were randomly located but demographic quotas were set in order to mirror the national population. A target of at least 35 respondents was set for each of the 9 versions of the questionnaire.

The data were obtained from interviewer-led completion of questionnaire. Participants were recruited by the data collecting agency from convenient locations and were not paid for participation. Interviewers were given clear instructions on administering the questionnaire. First, general information was given about risks and severity of both infectious diseases and their hypothetical vaccines. This was followed by a description of three hypothetical vaccines and an explanation of the DCE questions. Subsequently, respondents were presented with the questionnaire. The questionnaire contained the 13 choice questions explained above, as well as questions about the respondent’s knowledge, awareness and beliefs regarding vaccination against infectious disease in general, about the sources of information regarding child vaccination related issues that are known to them, and about socio-demographic attributes. Their awareness of infectious disease was measured from their perceptions of the severity of rotavirus infection. They were also asked if they believe that ‘immunisation weakens a child’s natural immunity’, their response to this statement was taken as a proxy of their belief about immunisation.

We used a logistic regression model to analyse the data as the dependent variable was binary- 1 for vaccination and 0 for no vaccination. Since respondents have to make several choices, the data have a panel structure. To account for the potential correlation between the responses given by each respondent, a random effects model was applied. The explanatory variables were the probabilities and severities of VAAE and of infection and the price of the vaccine. Because the different diseases vary considerably in terms of their probability and severity, the effects of such variation may not be adequately captured by the estimated parameters of risk and attributes. Two dummy variables were therefore included representing the diseases - NIPD and IPD.

The variable representing the severity of the health states was the corresponding visual analogue scale (VAS) score given to it by the respondent, rescaled by subtracting it from 100 so that severity reflects a decrement from full health, so that 100 is the worst state possible and 0 the best. As noted earlier, participants were asked to rate 6 of the 15 states that appear in the choice sets. For the health states that participants did not rate, the VAS scores were imputed using the mean imputation method. Mean imputation is the replacement of a missing observation with the mean of the non-missing observations for that variable. Although such simple imputation methods are commonly used in the social sciences [26], [27] they are often not adequate to handle the missing data problem [28]. The sensitivity of the regression results were checked using an alternative imputation approach. In the alternative imputation method missing values were imputed from the distribution of health state values observed in the non-missing observations.

Marginal effects of the attributes on the probability of vaccination were estimated using the Stata program *margeff*, which calculates the average marginal effects for each observation and averages these across all observations. The monetary valuation of each attribute or price is given by the trade-offs between price and each attribute that are implicit when respondents make choices [18], [19]. The discrete choice experiment is designed to replicate partly the decision making process when respondents choose either to vaccinate or not by making trade-offs between the attributes. The trade-offs are quantified by dividing each regression coefficient by the regression coefficient for price. This gives the equivalent amount of income respondents are prepared to give up or accept for a change in the level of another characteristic. Confidence intervals for the WTP/WTA ratio were calculated using the Krinsky-Robb method using 2,000 replications [29].

## Results

369 participants completed a questionnaire, yielding 4,753 individual choice observations. Table 1 compares the socio-demographic profile of the sample with that of the relevant UK population i.e. mothers of children under the age of 5 years. The sample is slightly over-represented by people from skilled and unskilled manual worker groups and underrepresented from supervisory and managerial socioeconomic groups. The sample is also relatively old and has a lower level of education.

**Table 1:** Characteristics of respondents and corresponding population statistics.

	Sample (N = 369)	Population		Sample (N = 369)	Population
**Age**		*	**Education**		***
under 20	4.1%	6.8%	Degree or equivalent	13.6%	26.30%
20–29	42.2%	44.9%	Higher education below degree	14.4%	15.19%
30–39	43.2%	44.8%	A level equivalent	14.6%	15.19%
40 and over	10.5%	3.5%	GCSE/O level equivalent	37.4%	24.72%
Missing	0.8%	–	CSE other grade equivalent	4.6%	8.39%
**Region**		**	None	14.9%	7.71%
North East	5.6%	5.7%	Missing	0.5%	–
North West	16.0%	15.3%	**Socioeconomic status**		****
Yorks	9.1%	11.5%	Managerial	19.8%	28.02%
West Midlands	9.1%	12.0%	Supervisory/clerical	21.7%	28.70%
London	11.0%	16.7%	Skilled manual	21.7%	20.02%
South East	5.6%	9.5%	Unskilled manual	34.4%	23.06%
South West	10.0%	11.4%	Missing	2.4%	-
Wales	22.3%	6.6%			
Scotland	11.3%	11.4%			

* Office of National Statistics [30];

** Office of National Statistics [31];

*** Health Survey for England [32];

**** Office of National Statistics [33].

347 respondents (94%) chose vaccination in the choice set where vaccination was most attractive. 118 respondents (31.9%), always made the same choice to vaccinate or not to vaccinate irrespective of the different levels of the attributes, of whom 107 (28.9%) always chose vaccination.

Aggregating the stated choices of all respondents over all choice sets we find that the vaccination rate for all vaccines together is 77%, with the highest rate (91%) being observed in case of Invasive-Pneumococcal disease, and the lowest rate (68%) observed for Rotavirus disease.

Table 2 shows the parameter estimates from the baseline logit model. The Likelihood-ratio test shows that it is preferable to use panel rather than pooled estimates and the Hausman test (*χ^2^*(7) = 2.73, p = 0.90) shows that a random rather than fixed effects model is preferable. The fraction of correctly classified outcomes (78%) suggests that the model is a reasonable fit to the data.

**Table 2:** Baseline regression model.

Parameter	coefficient	Standard error	P>|z|	Marginal effect
Price	−0.003	0.001	0.019	0.000
Probability of VAAE	−0.066	0.059	0.265	−0.007
Probability of disease	0.002	0.001	0.004	0.000
Severity of VAAE	−0.041	0.003	0.000	−0.004
Severity of disease	0.023	0.004	0.000	0.003
NIPD	0.603	0.245	0.014	0.082
IPD	1.991	0.286	0.000	0.195
Constant	1.836	0.368	0.000	
ρ (standard error)	0.57 (0.032)			
Log Likelihood	−1391.31			
?^2^ (p)	581.73 (0.000)			
Correct prediction	78.13%			
N	3660			

VAAE: vaccine associated adverse events; NIPD: non−invasive pneumococcal disease; IPD: invasive pneumococcal disease.

The estimated coefficients all have the expected signs, and all attributes other than the probability of VAAE are significantly different from zero at the 5% level. Respondents are more likely to vaccinate their child with a higher probability of disease, a higher level of disease severity, a lower level of severity of VAAE and a lower price. Compared with rotavirus, they are also more likely to vaccinate if the disease is NIPD and even more so if the disease is IPD.

Table 3 shows the estimates from the extended model, in which the effect of regret is included. Both regret variables are significant, with the theoretically expected sign. In general, respondents anticipate much higher regret from a decision not to vaccinate than to vaccinate. We can estimate what might be called ‘regret elasticities of demand’; if anticipated regret from deciding to vaccinate increases by one unit, then the propensity to vaccinate decreases by 2.8%, whilst a one unit change in regret from deciding not to vaccinate increases the probability of a child being vaccinated by 5.9%. In the extended model, the severity of health effects associated with infection and VAAE both exert a statistically significant effect on demand, with the expected signs, but the probabilities of both infection and VAAE fail to achieve statistical significance.

**Table 3:** Extended (Baseline plus Regret) model.

Parameter	coefficient	Standard error	P>|z|	Marginal effect
Price	−0.002	0.005	0.707	0.000
Probability of VAAE	0.262	0.160	0.102	0.022
Probability of disease	0.002	0.001	0.135	0.000
Severity of VAAE	−0.044	0.008	0.000	−0.004
Severity of disease	0.028	0.009	0.001	0.002
Anticipated regret vaccination	−0.288	0.062	0.000	−0.024
Anticipated regret not vaccinated	0.591	0.074	0.000	0.049
NIPD	0.360	0.591	0.548	0.034
IPD	1.466	0.681	0.031	0.120
Constant	−1.295	0.998	0.194	
ρ (standard error)	0.66 (0.046)			
Log Likelihood	−411.88			
?^2^ (p value)	106.94 (0.000)			
Correct prediction	78.61%			
N	1093			

VAAE: vaccine associated adverse events; NIPD: non-invasive pneumococcal disease;

IPD: invasive pneumococcal disease.

Table 4 shows estimates of WTP and WTA derived from the baseline model. Those with a positive coefficient are WTP, those with a negative coefficient are WTA. We have also checked WTP/WTA estimates where the alternative to mean imputation was applied to health states that participants did not rate The regression coefficients are reported in Table 5. The estimated coefficients are sensitive to the imputation method, and leads to a statistically insignificant price coefficient and therefore WTP estimates are also insignificant. Sensitiveness of estimates to the alternative imputation method led us to choose the mean imputation over the distributional imputation method.

**Table 4:** Estimates of willingness to pay or willingness to accept in £.

Parameter	WTP/WTA	Upper & lower Confidence Interval
Probability of VAAE	−19.39	−109.49, 15.42
Probability of disease	0.52	0.08, 2.55
Severity of VAAE	−12.15	−46.71, −6.14
Severity of disease	6.91	3.29, 27.39
NIPD	178.29	15.39, 867.38
IPD	588.44	247.11, 2483.43

VAAE: vaccine associated adverse events; NIPD: non-invasive pneumococcal disease; IPD: invasive pneumococcal disease; WTP: willingness to pay; WTA: willingness to accept.

**Table 5:** Baseline regression results adopting alternative imputation method.

Parameter	coefficient	Standard error	P>|z|	Marginal effect
Price	−0.002	0.001	0.139	0.000
Probability of VAAE	−0.077	0.056	0.172	−0.009
Probability of disease	0.002	0.001	0.000	0.000
Severity of VAAE	−0.025	0.002	0.000	−0.003
Severity of disease	0.010	0.002	0.000	0.001
NIPD	0.784	0.233	0.001	0.126
IPD	2.517	0.264	0.000	0.264
Constant	1.446	0.315	0.000	
ρ (standard error)	0.53 (0.033)			
Log Likelihood	−1469.30			
?^2^ (p)	530.89 (0.000)			
Correct prediction	77.67%			
N	3660			

VAAE: vaccine associated adverse events; NIPD: non-invasive pneumococcal disease;

PD: invasive pneumococcal disease.

Table 6 shows estimates of WTP/WTA values according to different socio-demographic variables. The socioeconomic status of households was categorized in two groups – supervisory and managerial households are combined in one group representing higher socioeconomic status, and households that have skilled and unskilled manual workers are combined in another group representing lower socioeconomic status. Lower income and socioeconomic status mothers have a relatively high WTP to avoid disease but a higher WTA to accept severe VAAE. Education and ethnicity of mothers had no influence on WTP/WTA.

**Table 6:** Estimates of willingness to pay or willingness to accept (in £) by different socio-demographic characteristics.

	Vaccine probability	Diseaseprobability	Vaccine severity	Disease severity	NIPD	IPD
**Socioeconomic status**						
Supervisory & managerial	21.94	0.47	−11.15	6.52	81.84	466.67
Skilled & unskilled manual worker	−60.33	0.73	−14.26	8.59	314.12	773.37
**Perceived disease severity**						
Low	−50.45	0.82	−14.63	9.08	323.60	847.98
High	14.97	0.23	−10.31	4.78	25.13	335.17
**Income**						
<£25,000	−40.21	1.35	−35.94	19.02	296.94	1614.97
>£25,000	15.11	0.51	−10.53	5.49	131.77	504.29
**Education**						
A level & above	−5.40	0.55	−12.46	6.88	155.82	578.53
GCSE or below	−31.69	0.51	−12.15	7.05	207.46	617.78
**Ethnicity**						
Non-white	−10.23	−0.58	7.17	−7.07	131.18	5.43
White	−16.83	0.46	−11.21	6.42	171.54	557.76
**Immunisation weakens immunity**						
Disagree	−23.94	0.47	−15.36	8.05	154.02	616.43
Agree	4.42	0.16	−2.66	5.31	70.95	150.03

NIPD: non-invasive pneumococcal disease; IPD: invasive pneumococcal disease.

Mothers’ awareness is proxied by their perceptions of the severity of rotavirus infection. Mothers’ awareness is categorised into two – very serious and fairly serious has been grouped as high awareness and the low awareness group includes perceived severity of serious, not very serious and not at all serious. Mothers who perceive rotavirus infection to be serious (i.e., higher awareness) have a relatively high WTA for VAAE. Similarly, mothers with a higher awareness of the risk of infectious disease are willing to pay more to avoid the disease. Mothers who disagreed with the statement that ‘immunisation weakens a child’s natural immunity’ had a higher WTP to avoid the disease and higher WTA for the VAAE.

Table 7 shows the probability of vaccination predicted from the baseline regression model at different levels of attributes. Price and probability attributes had 3 discrete levels, but health severity is measured on a continuous scale (0–100 scale). For ease of reporting, we have grouped the severity of the health variable into 3 different arbitrary levels: low if the severity of the health state is <40, medium if the severity of the health state is greater than 40 but less than 70, and high if the severity of the health state is above 70.

**Table 7:** Predicted probability of vaccination at different levels of attributes.

	Rotavirus	NIPD	IPD
**Cost of vaccine**			
Low	0.87	0.84	0.98
Medium	0.75	0.78	0.97
High	0.76	0.81	0.97
**Probability of VAAE**			
Low	0.76	0.83	0.98
Medium	0.78	0.81	0.97
High	0.85	0.79	0.97
**Probability of disease**			
Low	0.70	0.81	0.97
Medium	0.78	0.80	0.97
High	0.88	0.82	0.98
**Severity of VAAE**			
Low	0.91	0.89	***0.99***
Medium	0.56	0.67	0.95
High	0.45	***0.41***	0.81
**Severity of disease**			
Low	0.59	0.69	0.80
Medium	0.81	0.85	0.95
High	0.88	0.94	0.98

VAAE: vaccine associated adverse events; NIPD: non-invasive pneumococcal disease;

IPD: invasive pneumococcal disease.

These predicted values show that the demand for vaccination increases with the severity of disease, and is negatively related to the severity of VAAE. The price of vaccine has a negative influence on vaccine uptake, but the effect of cost is not monotonically decreasing in some price ranges. The predicted probability of vaccination is highest against IPD when the vaccine has low severity VAAE, and the lowest probable uptake arises for NIPD when vaccine leads to high severity VAAE. Overall, the predicted probabilities are higher for vaccination against IPD, followed by NIPD and rotavirus.

## Discussion

Our results demonstrate that the perceived severity of the disease and the perceived severity of VAAEs influence mothers’ decision making about vaccination of their children. Socioeconomic status, income, and awareness and beliefs regarding vaccination were all found to be important influences on the choice of whether or not to vaccinate. These findings add to other research into attitudes towards immunisation [1], [15], [17], [34].

Our results suggest that anticipated regret is also an important determinant of choices about vaccination. This adds to the findings of Connolly and Reb [24], who showed a correlation between regret and the decision to vaccinate. Similarly, Gallagher and Povey [15], in exploring the effect of anticipated regret on the intention to vaccinate against influenza, found that emotional feelings significantly increased the intention to vaccinate. Specifically, the influence of regret appears to be asymmetric – decisions are more sensitive to *ex ante* regrets about not vaccinating than they are to regrets about vaccinating. We have no information about why this is so, but one possible reason is that the dominant societal point of view, reinforced by pro-vaccination messages from health professionals, is that vaccination should be the norm. Regret derived from the negative consequences of deviating from a norm may be much greater than that from adhering to one.

The results show that anticipated regrets are strong predictors of vaccination. The severities of health associated with both diseases and VAAEs are significant predictors of vaccination even when regret variables are included. But the coefficient for the probability of disease and VAAE were reduced and became statistically insignificant when regret variables are included (compared with the base case model). Anticipated regrets may have mediated the relationship between the probability of disease/side effects and vaccination, but such relationship could not be inferred from the current findings.

Social gradients with respect to social grade, income, awareness, and beliefs of mothers influence the decision to vaccinate. The findings suggest that parents with a high social status are less willing to pay to avoid the severity of disease, and require less compensation to accept the severity of vaccine side effects. Similar effects have been observed with respect to household income. Intuitively it seems likely to be due to an income effect as parents with higher social status may shift away to safer vaccines. This kind of behaviour is observed in the case of measles, mumps and rubella (MMR) vaccines where parents of higher income group tended to prefer single doses of private MMR vaccines as opposed to combined MMR vaccine which had alleged (although unproven) side effects [35].

The results also show that mothers who have a high awareness about the risk of infectious diseases require more compensation to accept vaccine side effects than mothers who have less awareness. Similarly, mothers with a high awareness of the risk of infectious diseases are willing to pay more to avoid the disease compared to mothers with a low awareness. Similar results are observed with respect to mothers’ belief about immunisation. Mothers who disagree with the statement that ‘immunisation weakens a child’s natural immunity’ have consistently shown a higher WTP to avoid the disease and a higher WTA for avoiding vaccine side effects. The reasons could be that mothers who are more aware of disease are also more responsive to vaccine side effects, but the actual mechanism of such behaviour could not be explored here.

These findings have implications for both policy and research. Previous authors have attributed the decline in uptake in vaccination observed in many developed countries to the decline in the incidence of disease [36]–[41]. Our findings do not support this hypothesis, as mothers’ decisions to vaccinate their children do not appear to be very sensitive to the probability of disease, or indeed VAAE. Instead, mothers appear to be more sensitive to the severity of infection and VAAE. This leads to a number of implications. First, declines in vaccine coverage should be more strongly associated with a change in the perceived severity of VAAE, as happens with a vaccine scare, than with declines in disease incidence. Secondly, public health messages should put corresponding emphasis on severity to enable people to make better choices in accordance with their preferences. Thirdly, studies of decisions to vaccinate should take the perceived severity of the health states into account.

In addition, anticipated regret appeared to play a significant role in mothers’ decision-making. Perhaps the asymmetry in ‘regret elasticities’, which suggest that demand is more sensitive to anticipated regret from the child acquiring the disease than VAAEs, has helped to keep vaccine coverage relatively high, despite steep declines in disease incidence. Further research in this area appears warranted.

The success of vaccination programmes relies as much on the willing and active participation of mothers as it does on the availability of safe and effective vaccines. A greater focus on the factors affecting the demand for vaccines may bring significant pay-offs in terms of improved population health as well as the efficiency and equity of these large-scale programmes.

## Supporting Information

Table S1 Discrete Choice Experiment (DCE) attributes and their levels.
(DOCX)
