# Supplementary material for: The Effect of Perceived Risks on the Demand for Vaccination: Results from a Discrete Choice Experiment
Source: PLoS One. 2013 Feb 8;8(2):e54149. doi: 10.1371/journal.pone.0054149 (PMC3568102; doi:10.1371/journal.pone.0054149)
Supplement: Table S1 — Discrete Choice Experiment (DCE) attributes and their levels. (DOCX) [file pone.0054149.s001.docx]

# Rotavirus Vaccine

|  | **Vaccinate** | **Don’t vaccinate** |
| --- | --- | --- |
| **Price** | 50, 100, 150 |  |
| **Probability** | **Probability of adverse effects**:  10 in 100000  20 in 100000  40 in 100000 | **Probability of infection**  10000 in 100000  20000 in 100000  50000 in 100000 |
| **Severity** | **Severity of adverse effects**  *Low*  Mild irritability (1 - 2 days)  Loss of appetite (1 - 2 days)  *Medium*  Mild irritability (3-6 days)  Loss of appetite (3-6 days)  Fever & fatigue (3-6 days)  *High*  Bowel obstruction (requires surgery to correct) | **severity of infection**  *Low*  Fever & abdominal pain (1-2 days)  Diarrhoea, but no dehydration (loss of body fluids) (1-2 days)  *Medium*  Fever & abdominal pain (3–5 days)  Vomiting and diarrhoea, leading to moderate dehydration (loss of body fluids) (3-5 days)  *High*  Fever & abdominal pain (6-9 days)  Vomiting and diarrhoea leading to severe dehydration (loss of body fluids) (6-9 days) |

# Non-invasive Pneumococcal Vaccine

|  | **Vaccinate** | **Don’t vaccinate** |
| --- | --- | --- |
| **Price** | 50, 100, 150 |  |
| **Probability** | **Probability of adverse effects**:  100 in 100000  200 in 100000  400 in 100000 | **Probability of infection**  500 in 100000  1000 in 100000  2000 in 100000 |
| **Severity** | **Severity of adverse effects**  *Low*  Mild irritability, redness and swelling at the site of injection  *Medium*  Mild irritability, redness and swelling (1-2 days)  Muscle pain and swelling of joints (1-2 days)  Mild fever (1-2 days)  *High*  Swelling of face, lips, and tongue (3-6 days)  Rashes, itching, and skin swellings (3-6 days)  Muscle pain and swelling of joints (3-6 days)  Mild fever (3-6 days)  Breathlessness (3-6 days) | **severity of infection**  *Low*  Mild cold and fever (1-2 days)  Breathing difficulties (1-2 days)  *Medium*  Heavy cold and fever (3-4 days)  Breathing difficulties (3-4 days)  Ear infection (ear discharge) (3-4 days)  *High*  Heavy cold and high fever (5 - 7 days)  Breathing difficulties (5 - 7 days)  Severe chest infection (5 - 7 days)  Ear infection (ear discharge) (5 - 7 days) |

# Invasive Pneumococcal Vaccine

|  | **Vaccinate** | **Don’t vaccinate** |
| --- | --- | --- |
| **Price** | 50, 100, 150 |  |
| **Probability** | **Probability of adverse effects**:  100 in 100000  200 in 100000  400 in 100000 | **Probability of infection**  20 in 100000  40 in 100000  60 in 100000 |
| **Severity** | **Severity of adverse effects**  *Low*  Mild irritability, redness and swelling at the site of injection  *Medium*  Mild irritability, redness and swelling (1-2 days)  Muscle pain and swelling of joints (1-2 days)  Mild fever (1-2 days)  *High*  Swelling of face, lips, and tongue (3-6 days)  Rashes, itching, and skin swellings (3-6 days)  Muscle pain and swelling of joints (3-6 days)  Mild fever (3-6 days)  Breathlessness (3-6 days) | **severity of infection**  *Low*  High fever  Blood poisoning  *Medium*  High fever  Blood poisoning  Brain damage (permanent disability)  Deafness (permanent disability)  Loss of vision (permanent disability)  *High*  High fever and brain damage leading to  Death |
